# Expression and Functional Studies on the Noncoding RNA, PRINS

**DOI:** 10.3390/ijms14010205

**Published:** 2012-12-21

**Authors:** Krisztina Szegedi, Anikó Göblös, Sarolta Bacsa, Mária Antal, István Balázs Németh, Zsuzsanna Bata-Csörgő, Lajos Kemény, Attila Dobozy, Márta Széll

**Affiliations:** 1 Department of Dermatology and Allergology, University of Szeged, Korányi fasor 6, H-6720 Szeged, Hungary; E-Mails: krisztinaszegedi@yahoo.com (K.S.); bacsasaci@msn.com (S.B.); nemeth.istvan.balazs@med.u-szeged.hu (I.B.N.); bata.zsuzsa@med.u-szeged.hu (Z.B.-C.); kemeny.lajos@med.u-szeged.hu (L.K.); dobozy.attila@med.u-szeged.hu (A.D.); 2 Institute of Plant Biology, Biological Research Center of the Hungarian Academy of Sciences, Temesvári krt 62, H-6726 Szeged, Hungary; E-Mail: antalmano@yahoo.com; 3 Dermatological Research Group of the Hungarian Academy of Sciences, University of Szeged, Korányi fasor 6, H-6720 Szeged, Hungary; E-Mail: szell.marta@med.u-szeged.hu; 4 Department of Medical Genetics, University of Szeged, Somogyi u. 4, H-6720 Szeged, Hungary

**Keywords:** noncoding RNA, nucleophosmin, keratinocyte proliferation, psoriasis, UV-B irradiation

## Abstract

PRINS, a noncoding RNA identified earlier by our research group, contributes to psoriasis susceptibility and cellular stress response. We have now studied the cellular and histological distribution of PRINS by using *in situ* hybridization and demonstrated variable expressions in different human tissues and a consistent staining pattern in epidermal keratinocytes and *in vitro* cultured keratinocytes. To identify the cellular function(s) of PRINS, we searched for a direct interacting partner(s) of this stress-induced molecule. In HaCaT and NHEK cell lysates, the protein proved to be nucleophosmin (NPM) protein as a potential physical interactor with PRINS. Immunohistochemical experiments revealed an elevated expression of NPM in the dividing cells of the basal layers of psoriatic involved skin samples as compared with healthy and psoriatic uninvolved samples. Others have previously shown that NPM is a ubiquitously expressed nucleolar phosphoprotein which shuttles to the nucleoplasm after UV-B irradiation in fibroblasts and cancer cells. We detected a similar translocation of NPM in UV-B-irradiated cultured keratinocytes. The gene-specific silencing of PRINS resulted in the retention of NPM in the nucleolus of UV-B-irradiated keratinocytes; suggesting that PRINS may play a role in the NPM-mediated cellular stress response in the skin.

## 1. Introduction

Psoriasis is a multifactorial, hyperproliferative, chronic inflammatory skin disease that affects 1%–3% of the adult Caucasian population (OMIM 177,900, www.ncbi.nlm.nih.gov), with a substantial negative impact on the patient’s quality of life [1]. Immunoregulatory abnormalities [2] as well as environmental and genetic factors [3,4] contribute jointly to the development of psoriasis. Hyperproliferation of the keratinocytes in the psoriatic plaques is triggered by infiltrating T-lymphocytes at the dermal–epidermal junction [5]. The keratinocytes of the uninvolved psoriatic epidermis are inherently oversensitive to proliferative signals, and this elevated level of sensitivity plays a crucial role in the development of psoriatic lesions [6,7]. Thus, resident skin cells and infiltrating immune cells cooperate in the formation of psoriatic lesions, but the exact molecular mechanisms that regulate the interactions between these cells are still far from understood. The identification of genes and proteins with altered expressions, either in the uninvolved or in the involved psoriatic skin, may therefore facilitate an understanding of the pathogenesis of this disease. While investigations of the molecular mechanisms behind psoriasis have identified numerous disease-associated genes and proteins [8–12], it is still unclear how the altered expressions of some genes contribute to the pathogenesis of psoriasis, how the differentially expressed molecules exert their action and what other molecules they may interact with.

We demonstrated earlier that PRINS, (psoriasis susceptibility-related RNA gene induced by stress), a noncoding RNA (ncRNA) first identified by our research group, is expressed more strongly in the uninvolved psoriatic epidermis than in the psoriatic involved or healthy epidermis, suggesting that the overexpression of PRINS in the uninvolved psoriatic epidermis may play a role in psoriasis susceptibility. Our *in vitro* experiments also revealed that PRINS functions as a regulatory RNA, playing a protective role in cells exposed to stress [13].

Since their first detection [14] numerous mRNA-like ncRNA transcripts have been found in different cell types. Besides alternative splicing and promoter-driven regulation, ncRNA molecules comprise a new level of regulation of protein-coding genes [15]. As yet few papers have provided partial or full descriptions of the structural complexes formed by ncRNAs and their protein partners [16–18], and their participation and roles in different cellular processes are still poorly understood. PRINS an RNA polymerase II transcribed RNA, is a 3681 nucleotide-long molecule. We originally described it in HaCaT keratinocytes where it participates in cellular stress response. BLAST searches (http://blast.ncbi.nlm.nih.gov/Blast.cgi) revealed that the PRINS gene is specific for anthropoid primates, its rodent ortholog could not be identified [13]. Our studies demonstrated a higher PRINS expression in the uninvolved psoriatic epidermis than in either the psoriatic involved or healthy epidermis [13]. These results suggest that the overexpression of PRINS in the uninvolved psoriatic epidermis may play a role in psoriasis susceptibility rather than in the precipitation of psoriatic lesions, and are in agreement with the previous observations that the keratinocytes of the uninvolved epidermis differ from healthy keratinocytes in their responses to external stimuli [6,19,20]. We additionally showed that PRINS may possess regulatory functions: the expression of the antiapoptotic G1P3 gene was found to be under the control of PRINS in various cell types [21].

We now report on the cellular and histological distribution of PRINS, determined by using *in situ* hybridization (ISH). Moreover, we present the results of an *in vitro* binding assay which suggests that PRINS interacts physically with the molecular chaperone protein nucleophosmin (NPM, B23) in HaCaT and normal human epidermal keratinocyte (NHEK) lysates and describe the first upregulation of this protein in psoriasis.

NPM is a nucleolar phosphoprotein [22] with a potential role as a positive regulator in cell proliferation [23,24]. A number of studies have shown that UV irradiation results in a rapid nucleoplasmic translocation of the otherwise predominantly nucleolar protein NPM in fibroblasts [25,26] and cancer cells [27,28].

Our experiments indicate a similar pattern of intracellular localization of NPM after UV-B exposure in cultured keratinocytes, and reveal that the gene-specific silencing of PRINS modifies the UV-induced intracellular shuttling of NPM suggesting that NPM is a physical and functional interactor of PRINS.

## 2. Results and Discussion

### 2.1. The Histological and Cellular Distribution of PRINS

By using real-time RT-PCR, we earlier demonstrated differences in PRINS expression in various human tissue samples [13]. For a more detailed analysis of PRINS expression we have now applied ISH. To investigate the pattern of PRINS expression pattern in different tissue types, we applied paraffin-embedded tissue chips containing thirteen sections from different healthy human organs on one slide. The samples were exposed to the same conditions during ISH, and this led to good comparability. The intracellular distribution of PRINS was also examined in cultured NHEKs.

**Figure 1:**
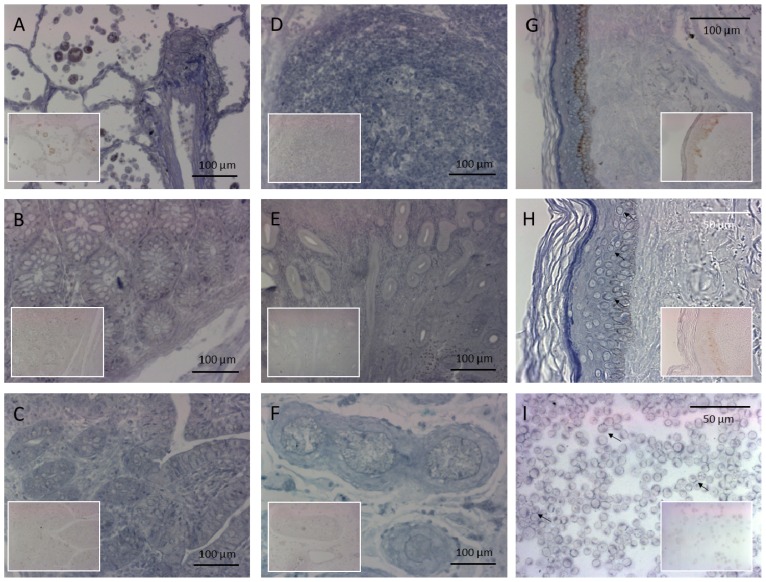
Detection of psoriasis susceptibility-related RNA gene induced by stress (PRINS) expression in various human tissue samples by ISH. The sections were incubated with an LNA RNA detection probe for PRINS and for the control staining (see insets at the same magnification) we used a scrambled control sequence. Relatively strong PRINS positivity was seen in the (**A**) lungs; (**B**) the large bowel; (**C**) the small bowel; (**D**) the lymph nodes; (**E**) the uterus; (**F**) the testicles; (**G**,**H**) the skin; and (**I**) cultured keratinocytes.

The ISH experiments demonstrated variable levels of expression in different human tissue samples: high levels of PRINS expression were detected in the gut, lungs, lymph nodes, uterus, testicles and skin (Figure 1A–H), whereas no staining was observed in the cerebrum and cerebellum (data not shown). The breast, kidney, stomach, and gallbladder tissue specimens displayed only moderate PRINS expressions (data not shown). These findings are in agreement with our previous results revealing variable PRINS gene expression levels in different healthy human organs. ISH demonstrated a relatively high PRINS positivity in the healthy epidermis.

The dermal and epidermal expression of PRINS significantly differed while a strong staining intensity in the epidermis was similar in the various layers. The stratum granulosum and stratum lucidum exhibited somewhat darker PRINS ISH staining (Figure 1G,H), which seemed to be specific, since the scrambled control probe did not result in the same staining intensity in these layers. There was only a moderate positivity in the dermis. In *in vitro* cultured keratinocytes (Figure 1I) exhibited strong nucleolar (indicated by small arrows) and perinuclear PRINS positivity and moderate homogeneous cytoplasmic staining. The cellular expression profile of PRINS is in agreement with the epidermal staining pattern.

### 2.2. PRINS Expression in Healthy and Psoriatic Skin

ISH experiments were performed to compare the expressions of PRINS in normal healthy (*n* = 10), psoriatic uninvolved (*n* = 6) and psoriatic involved (*n* = 6) skin samples. With a quantitative RT-PCR approach we earlier showed that PRINS was expressed more strongly in the psoriatic uninvolved epidermis than in either the normal or the involved epidermis [13].

**Figure 2:**
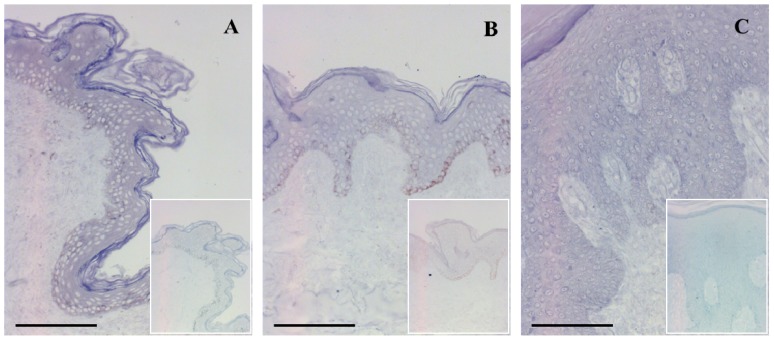
Detection of PRINS expression in psoriasis. ISH for PRINS expression was performed on healthy normal (*n* = 10), psoriatic uninvolved (*n* = 6) and psoriatic involved (*n* = 6) skin specimens. Sections were incubated with the PRINS-specific LNA probe or with the control scrambled (see insets at the same magnification) LNA probe. Representative stainings for each tissue types: (**A**) normal healthy skin; (**B**) psoriatic uninvolved skin; and (**C**) psoriatic involved skin. Bar = 100 μm.

The ISH results partially confirmed the Q-RT-PCR findings, indicating a moderately elevated level of PRINS expression in the uninvolved (Figure 2B) and involved (Figure 2C) epidermis relative to the healthy epidermis (Figure 2A). However, the differences in expression between the involved and uninvolved epidermis were not as pronounced as with the Q-RT-PCR approach. The explanation for this may lie in the different sensitivities of the two methods.

The differential distribution of PRINS (a multiple stress-related ncRNA) in the epidermal keratinocytes could be correlated with the abnormal keratinocyte functions seen in psoriasis and with the skin-relevant stress factors inducing the epidermal stress response.

### 2.3. *In Vitro* Identification of NPM as a PRINS-Interacting Protein

Our previous data suggested roles for PRINS in psoriasis susceptibility and the cellular stress response. It is also accepted that long mRNA-like ncRNAs form complexes with proteins [16–18], and act as regulators of various cellular functions [29].

**Figure 3:**
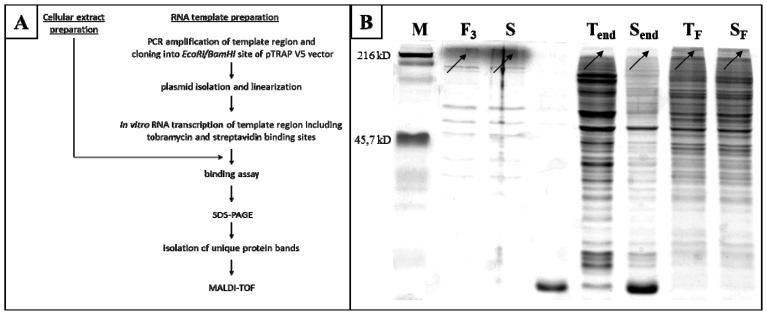
Identification of the direct interacting partner of PRINS. The *in vitro* binding assay and the consecutive experiments with the three independent cell lysates (two from HaCaT keratinocytes and one from cultured NHEK) were performed as depicted in the Figure. A 39mer RNA transcript of PRINS was used. The elutes obtained with the RNP-complex purification kit were run on SDS-PAGE. Bands (arrows) that appeared in the PRINS binding fraction but not for the control samples, were cut out from the gel and further analyzed with MALDI-TOF. (**A**) Flow chart of the *in vitro* experiments. (**B**) Following the binding assay in HaCaT cells, the proteins in the eluates were separated on 7.5% SDS-PAGE and stained with Coomassie Brilliant Blue. NPM from a ~250-kD band was identified by MALDI-TOF (shown by black arrows). S, elute from the 1st affinity matrix (tobramycin) bound with the *in vitro* transcribed PRINS and cellular extract. F_3_, elute from the 2nd affinity matrix (streptavidin) incubated with elute “S”. T_F_, flow-through from the tobramycin affinity matrix incubated with the cellular extract. S_F_, flow-through from the streptavidin affinity matrix incubated with the cellular extract. T_end_, elute from the tobramycin affinity matrix incubated with the cellular extract. S_end_, elute from the streptavidin affinity matrix incubated with the cellular extract

To investigate the molecular mechanisms involved in the stress-related functions of PRINS, we first searched for its intracellular counterparts. In an *in vitro* experiment (Figure 3A) we used a 39mer RNA transcript of PRINS; this region harbors the 19mer sequence of the gene successfully used for the gene-specific silencing of PRINS [13]. For the identification of PRINS-interacting proteins, *in vitro* transcribed RNA (PRINS AK676 sequence) was added to a tobramycin affinity matrix and the cell lysates were also added to the column. After several washing steps, the RNP particles were eluted from the tobramycin matrix and transferred to a streptavidin affinity matrix. Control elutes were taken from both the tobramycin and streptavidin columns, where the cellular extracts were added to the affinity matrix without the bound *in vitro* transcribed PRINS sequence. The elutes obtained with the RNP-complex purification kit were run on SDS-PAGE (Figure 3B). Bands (one with a molecular weight of >216 kD and another of ~50 kD) that appeared in the PRINS- binding fraction (S, F_3_) but not for the control samples (T_end_, S_end_) were cut out from the gel and further analyzed with matrix-assisted laser desorption/ionization time-of-flight (MALDI-TOF) mass spectrometer. The *in vitro* binding assay was performed and consecutive experiments with three independent cell lysates (two from HaCaT keratinocytes and one from NHEKs).

NPM was identified as a putative PRINS-binding protein in all three independent binding experiments. NPM, a multifunctional phosphoprotein, is implicated in mRNA processing [30,31] and also in the acute response of mammalian cells to environmental stress, when it stimulates DNA repair and reduces apoptosis [26]. It has been demonstrated that NPM forms oligomers involving highly conserved short loops, the ionic interactions between the monomeric subunits leading to the formation of a thermo-stable, chemically resistant pentamer; it has also been shown that NPM pentamers can form decamers *in vitro* by packing two pentamers on top of each other in a sandwich-like structure [32]. This explains why we identified NPM from a 250 kD band by MALDI-TOF and suggests a putative structure for the oligomer that is capable of housing long oligonucleotides such as long ncRNAs. Similar protein oligomer-RNA complexes have been documented previously such as the hexameric or heptameric complexes of Sm proteins that function as RNA chaperones and fulfill a number of central tasks in various types of RNA processing [33], and the bacterial Hfq protein that forms hexamers and also functions as an RNA chaperone complex [34]. The heterogeneous nuclear ribonucleoproteins (hnRNPs) play an active part in post-transcriptional gene regulation, e.g., RNA splicing and regulation of the stability and translation of target mRNAs. Most hnRNPs are primarily localized in the nucleoplasm, and can shuttle between the nucleus and the cytoplasm [35].

### 2.4. NPM Is Overexpressed in Psoriatic Involved Epidermis

**Figure 4:**
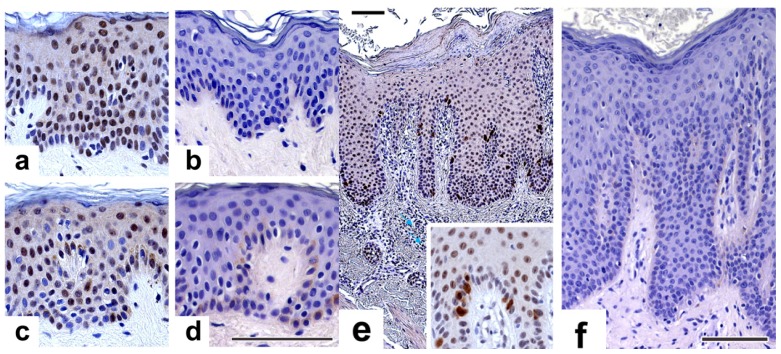
Immunohistochemical analysis of nucleophosmin (NPM) protein expressions in healthy and psoriatic skin samples. Paraffin-embedded tissues of healthy, non-lesional and lesional psoriatic skin samples were used for NPM-specific immunohistochemistry. After nonspecific antigen blocking, sections were incubated with anti-NPM mouse monoclonal antibody at a dilution of 1:500. The incubation with secondary antibody was followed by the development of a DAB color reaction, the intensity of which reaction was controlled under a light microscope. Sections were counterstained with hematoxylin. In the healthy and psoriatic uninvolved skin, the keratinocytes presented nuclear staining throughout all of the epidermal layers. In the healthy epidermis, some subgranular nuclei showed a high density of NPM, while in the uninvolved epidermis, a higher rate of spinous suprabasal nuclear staining was observed. In the psoriatic involved skin, the various layers of the epidermis exhibited a different NPM protein expression pattern. The keratinocytes presented pronounced cytoplasmic immunopositivity besides nuclear staining. Bar = 100 μm; (**a**) healthy epidermis; (**b**) isotype control for healthy epidermis; (**c**) psoriatic non-lesional; (**d**) isotype control for psoriatic non-lesional; (**e**) psoriatic lesional epidermis; (**f**) isotype control for lesional psoriatic epidermis.

The psoriasis-associated expression of PRINS has been clearly established by Sonkoly *et al.* [13]. We asked the question of whether NPM gave a characteristic pattern of staining that might correlate with the known overexpression of PRINS in the psoriatic uninvolved epidermis. NPM immunopositivity was apparent in all the studied skin samples. In both the healthy (Figure 4a) and the psoriatic uninvolved (Figure 4c) skin, the keratinocytes presented a nuclear staining throughout all of the epidermal layers. Although there were some subgranular nuclei that exhibited a high density of brown color in the healthy epidermis, a higher rate of positivity was noted among the spinous suprabasal keratinocytes than among the granular and subgranular keratinocytes in the uninvolved epidermis (Figure 4c). This finding is in agreement with previously reported ISH results [36] that indicated the highest accumulation of NPM transcript in the nuclei of epithelial cells. In the dermis, infiltrating immune cells and blood displayed showed pronounced nuclear NPM staining (small arrows). The cellular distribution of PRINS and that of NPM in the healthy epidermis showed similarities, supporting our results concerning their physical interaction.

In the psoriatic involved skin, the different layers of the epidermis exhibited different NPM protein expression patterns. In the extended spinous layer, only moderate NPM staining was detected, while in the basal layer and in the immediate suprabasal layer NPM was expressed at high levels. The most interesting phenomenon was seen in the basal layer of the psoriatic involved epidermis (Figure 4e), where certain nests of dividing keratinocytes presented marked cytoplasmic immunopositivity besides nuclear staining. This staining pattern finally resulted in a very interesting “speckled” overall image in the psoriatic involved epidermis. This finding is in accord with the results of Amin and co-workers who recently reported that NPM is localized at the chromosome periphery during mitosis [37], when the nuclear membrane is demolished. We hypothesize that the interesting “speckled” staining pattern of the basal keratinocytes in the involved psoriatic epidermis reflects this phenomenon.

In a previous study of the expression of NPM, exclusive nuclear staining was seen in squamous cell carcinoma, whereas in basal cell carcinoma samples a certain level of cytoplasmic NMP immunopositivity was also apparent [38]. These observations suggest that NPM may play a role in normal keratinocyte proliferation and its overexpression is a key motive in certain hyperproliferative and malignant skin diseases.

### 2.5. NPM Expression Decreases as Keratinocytes Differentiate

**Figure 5:**
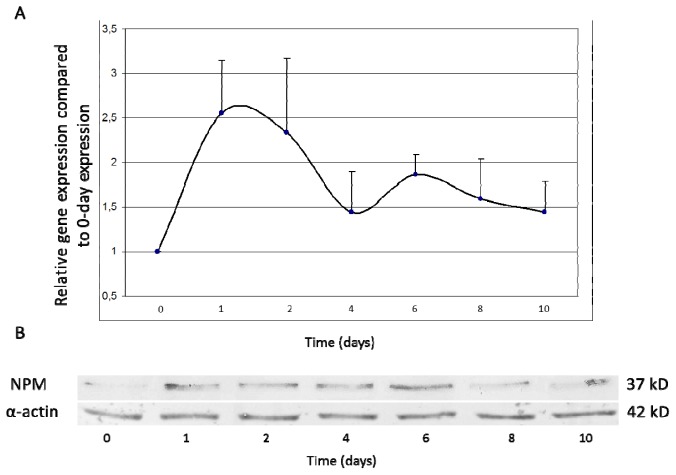
Expression of NPM protein during keratinocyte proliferation/differentiation *in vitro*. Cultured normal human epidermal keratinocyte (NHEK) cultures (*n* = 3) were grown until subconfluency, when the 0-h sample was taken. (**A**) Changes in the expression of NPM mRNA were analyzed by real-time RT-PCR at the indicated times. Relative expression is shown compared to the 0-h sample. (**B**) At the indicated times, cells were harvested and lysed, then subjected to denaturing SDS-PAGE analysis and electroblotted to nitrocellulose. NPM protein was detected with a mouse monoclonal anti-NPM antibody and visualized with the NBT/BCIP system.

Since the epidermal keratinocytes were found to express NPM at high levels, we investigated whether this expression was dependent on the proliferation/differentiation state of the keratinocytes. In a set of *in vitro* experiments on spontaneously differentiating 3rd passage NHEKs, the process of differentiation was followed via the increase in a differentiation marker (keratin 10) and the decrease in a proliferation-related marker (alpha5 integrin) (Figure S1). The 0-h samples were taken from subconfluent cultures. Real-time RT-PCR experiments (*n* = 3) (Figure 5A) and Western blot experiments (*n* = 3) (Figure 5B) revealed that the NPM mRNA and protein expressions were highest on days 1 and 2 and then gradually declined during the 10-day follow-up.

The transcriptional level upregulation of NPM occurred in proliferating cells and, although a slight decline in NPM level was observed, the protein remained at a high level in the less proliferating, more differentiated keratinocytes when the alpha5 integrin was already decreasing [39], suggesting that the NPM protein is relatively stable once expressed in the cells. The fact that the mRNA level decreased rapidly (day 4), while the protein level in the keratinocytes declined in a prolonged, more moderate manner (day 8), indicates that posttranscriptional, translational regulation and posttranslational modifications such as phosphorylation during the maturation and processing of the protein might play important roles in the NPM protein expression in keratinocytes. NPM is known to be phosphorylated by protein kinase CK2 in the interphase [40] and by p34^cdc2^ kinase during mitosis [41,42]. It has also been shown that the level of NPM phosphorylation correlates with the cellular proliferative activity and is enhanced at mitosis [43–46]. However, a precise determination of exactly which phosphorylation pathway(s) play(s) a role in NPM stabilization in the keratinocytes requires further experiments.

### 2.6. Intracellular Localization of NPM Protein in Keratinocytes

NPM is a ubiquitously expressed nuclear phosphoprotein which shuttles continuously between the nucleus and the cytoplasm [47]. Within the nucleus, NPM is predominantly localized in the nucleoli, with its highest levels in the granular component that contains the more mature preribosomal particles [22], but a significant fraction of NPM can also be detected in the nucleoplasm. NPM is a multifunctional protein with multiple locations in the cell. During the cell cycle, NMP is dynamically localized [37] and it is also redistributed from the nucleolus in response to cytotoxic drugs and genotoxic stress. In fibroblasts and cancer cells, UV irradiation results in a rapid nucleoplasmic translocation of the nucleolar NPM [25,27]. To investigate whether we could observe a similar intracellular localization of NPM in keratinocytes after UV-irradiation, we applied an immunocytochemistry method. In these experiments, we used a newly established keratinocyte cell line, HPV-Ker [48]. The UV responses of NHEKs and HPV-Ker cells were compared. The viability and the expression of several UV-induced genes were identical in the two cell types. Moreover, the stress-induced p53 gene expression in the HPV-Ker cells presented the same UV-B inducibility as that in the NHEKs. The functional similarities between the NHEKs and the HPV-Ker cells suggested that HPV-Ker cells are good model cells for the study of UV-induced keratinocyte functions.

**Figure 6:**
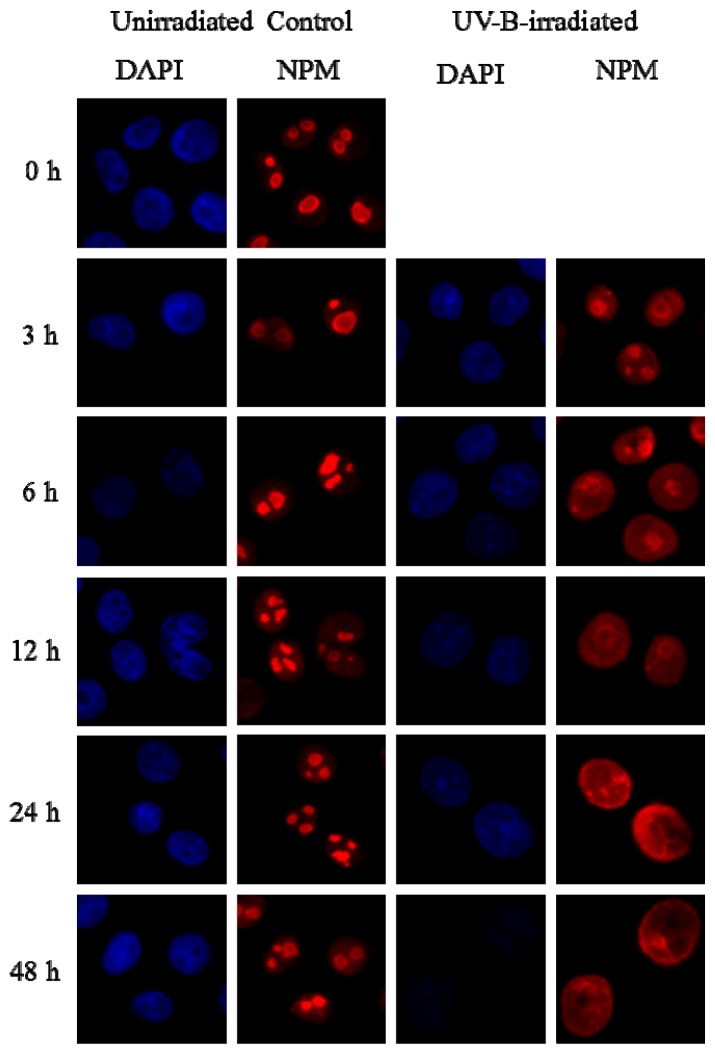
UV-B radiation induces the intracellular trafficking of nucleoplasmic NPM in HPV-Ker cells. Unirradiated and UV-B-irradiated (312 nm, 40 mJ/cm^2^) HPV-Ker cells were followed for the indicated periods of time, fixed and immunostained for NPM. DNA was stained with 4,6-diamidino-2-phenylindole (DAPI).

UV-B exposure caused a relocalization of NPM from the nucleolus to the nucleoplasm in HPV-Ker cells (Figure 6). The UV-B-induced subcellular shuttling could be detected quite early after UV-B irradiation: 3 h after treatment, slight translocational changes were observed. The immunofluorescence in the nucleoplasm was most increased at 12 and 24 h. Forty-eight hours following UV-B treatment, the protein had mostly returned to the nucleolus. The time course studies showed that the stimulation of NPM shuttling by UV-B irradiation was a rapid and transient process. The intracellular localization pattern of NPM after UV-B exposure was similar in both the cultured NHEKs (Figure S2) and the HPV-Ker cells, which suggests that HPV-Ker cells could be appropriate models for further studies of intracellular NPM trafficking.

### 2.7. PRINS Modifies the UV-B Irradiation-Induced Intracellular Shuttling of NPM

To determine whether PRINS had any effect on NPM intracellular trafficking, we silenced the expression of PRINS in growth factor-deprived HPV-Ker cells with a vector-based method [13] and studied the NPM shuttling under normal conditions and after UV-B exposure. NPM shuttling was compared in HPV-Ker cells transfected with a specific PRINS-silencing construct (AK696) and in HPV-Ker cells transfected with a control construct (SC1313). Real-time RT-PCR revealed successful gene-specific PRINS silencing (Figure S3A), which resulted in a moderate induction of NPM expression (Figure S3B).

**Figure 7:**
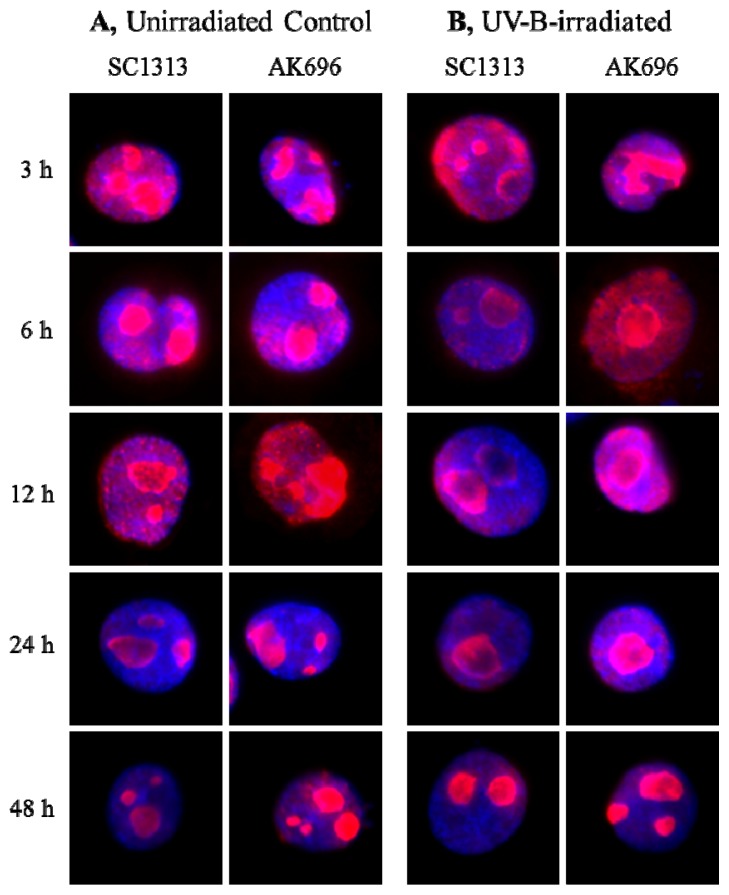

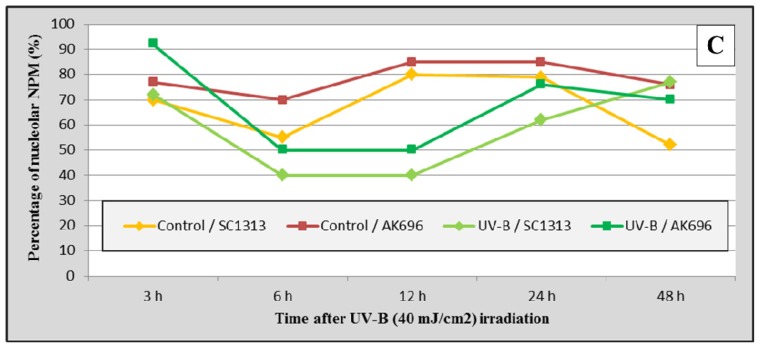
Silencing of the PRINS expression modifies the UV-B-induced trafficking of NPM. HPV-Ker cells were transfected with a PRINS gene-specific silencing vector (AK696). Control cells were transfected with a vector containing a scrambled sequence (SC1313). One day after the transfection, the unirradiated (**A**) and the UV-B-irradiated (**B**) HPV-Ker cells were followed for the indicated periods of time, fixed and immunostained for NPM (red). DNA was stained with DAPI (blue). Following the immunostaining, we performed a semiquantitative analysis of the cells. In every group, 25 fields of view were counted. Mean values are plotted on the chart (**C**).

In untreated samples, the NPM was localized mostly within the nucleolus in both the SC1313 and the AK696-transfected cells (Figure 7A). The relocalization of NPM from the nucleolus to the nucleoplasm in the control SC1313-transfected cells at the indicated times after UV-B exposure was similar to our preliminary observations in HPV-Ker cells. As compared with the control cells, the AK696-transfected cells (Figure 7B) showed a moderate retention of NPM in the nucleolus following UV-B treatment. To validate the immunocytochemical observations, a semiquantitative analysis was performed (Figure 7C). The nuclear transition of NPM was inhibited in AK696-transfected cells after UV-B irradiation. Interestingly, we observed that the transfection itself slightly modified the shuttling of NPM even in the UV-B negative group.

Besides the above-described NPM immunocytochemical detection, the shuttling of the protein was also studied by using an NPM-GFP chimeric construct. The results of the two experimental approaches were identical: both suggested that PRINS contributes to the UV-B-induced intracellular trafficking of NPM (Figure S4).

Our data indicate that PRINS does not act alone, but forms a complex with the NPM protein and contributes to its stress-related intracellular trafficking.

### 2.8. *In Silico* Prediction and Gene-Specific Silencing Experiments Suggest that PRINS Might Be Part of the Transcription Regulatory CTCF/NPM Complex and Acts as a Negative Regulator of Cell Cycle Progression

It has recently been demonstrated that NPM is a major interacting protein of the zinc finger protein CCCTC binding factor (CTCF), which is known to be a critical mediator of multiple epigenetic processes [49]. The gene-specific silencing of NPM causes severe mitotic defects and delayed mitotic progression, suggesting a crucial regulatory role in cell proliferation [37].

Interestingly, when we performed an *in silico* prediction, transcriptional regulators with 11 highly conserved zinc finger domains were among the potential PRINS-binding proteins and the NPM-interacting CTCF binding factor belongs in this zinc finger protein family (Table S1).

CyclinD1, a known target gene of the NPM/CTCF factor regulatory complex, is upregulated in psoriasis [50]. In our preliminary experiments, the gene-specific silencing of PRINS resulted in a substantial induction of cyclin D1 expression (data not shown). Moreover, we also showed that the silencing of PRINS resulted in a slight elevation of NPM expression (Figure S3B). This finding is in good agreement with the differentiation-related expression of PRINS: it is expressed most strongly in serum-starved, contact-inhibited HaCaT keratinocytes and its expression decreases as the cells start to proliferate after release from cell quiescence; moreover, PRINS is strongly expressed in the uninvolved psoriatic epidermis and its expression is substantially downregulated in the hyperproliferative involved epidermis [13]. These data indicate that PRINS might be part of the transcription regulatory CTCF/NPM complex, and as part of this molecular complex it plays a negative regulatory role in the cell cycle progression. The involvement of long ncRNAs in molecular regulatory complexes has already been documented in the literature [16].

## 3. Experimental Section

### 3.1. Cell Cultures

HaCaT keratinocytes, the immortalized human keratinocyte cell line kindly provided by Dr. Fusenig, N.E. (Heidelberg, Germany), was cultured to subconfluency in T75 tissue culture flasks (Corning Incorporated, Corning NY USA) and maintained in keratinocyte serum-free medium (Gibco^®^ Keratinocyte SFM Kit; Life Technologies, Copenhagen, Denmark) supplemented with 1% antibiotic/antimycotic solution (PAA, Pasching, Austria) and 1% l-glutamine (PAA, Pasching, Austria) at 37 °C in a humidified atmosphere containing 5% CO_2_. The medium was changed every two days. Cells were synchronized as described previously [39].

NHEKs were obtained from normal human epidermis and cultured as described earlier [51]. Briefly, NHEKs were separated from a skin specimen obtained from the Plastic Surgery Unit of our Dermatology Department. The epidermis and the dermis were separated by overnight incubation in Dispase (Roche Diagnostics, Manheim, Germany), and the keratinocytes were obtained after a maceration in 0.25% trypsin. Cells were maintained in keratinocyte serum-free medium (Gibco^®^ Keratinocyte SFM Kit; Life Technologies, Copenhagen, Denmark), which contains EGF and BPE as supplements. The calcium concentration of the medium is <0.1 mM. For the experiments 3rd passage keratinocytes were used. In our *in vitro* differentiating model, fresh medium was added to subconfluent 3rd passage keratinocytes and samples were taken from the culture at the indicated time points. The rapid upregulation of alpha5 integrin on day 1 indicated intensive keratinocyte proliferation and, as the culture became confluent on day 2, the expression of alpha5 integrin gradually decreased (Figure S1).

The HPV-Ker cell line was immortalized by the HPV E6 oncogene as described previously [48]. HPV-Ker cells were grown in 75 cm^2^ cell culture flasks and maintained in keratinocyte serum-free medium (Gibco^®^ Keratinocyte SFM Kit; Life Technologies, Copenhagen, Denmark) supplemented with 1% antibiotic/antimycotic solution (PAA, Pasching, Austria) and 1% l-glutamine (PAA, Pasching, Austria) at 37 °C in a humidified atmosphere containing 5% CO_2_. The medium was changed every two days.

Cells were subjected to UV-B irradiation (312 nm, 40 mJ/cm^2^) in phosphate-buffered saline (PBS; 153 mM NaCl, 7.67 mM Na_2_HPO_4_, 2.67 mM NaH_2_PO_4_) at room temperature (RT). Immediately after irradiation, the PBS was aspirated off, and culture medium was added to the culture dishes. Cells were harvested at the indicated times after UV-B exposure.

### 3.2. *In Situ* Hybridization

Custom LNA mRNA detection probes for PRINS with the sequence 5′-3′: /5DigN/AAGCTTCTGTCCTCATTAGTCTTC/3Dig_N/ and scrambled control sequence 5′-3′: /5DigN/AAGCCTCCGTTCTTATTAGTCTTC/3Dig_N/ were ordered from Exiqon (Exiqon A/S, Vedbaek, Denmark).

Tissue chips: Formaldehyde-fixed blocks measuring 4 mm in diameter of normal breast, cerebellum, cerebrum, gallbladder, kidney, large bowel, small bowel, lung, lymph node, skin, stomach, testicle and uterus samples were placed into a tissue microarray (TMA) block. The fixation of samples was standardized (4% buffered formaldehyde for 24 h at RT, in a volume of 1:10). After paraffin embedding, the TMA was cut into 4-μm-thick sections, which were rehydrated with increasing concentrations of alcohol.

HPV-Ker cells were trypsinized and harvested by centrifugation, and resuspended in PBS. One hundred thousand cells were centrifuged onto a slide by using a cytocentrifuge (Cytopro™, Wescor, Logan, UT, USA) and dried overnight at RT. The slides were fixed in 2% paraformaldehyde for 20 min at RT.

After 2 × 5-min PBS washes, tissue chip slides and slides with HPV-Ker cells were treated for 15 min and for 5 min, respectively, with 20 μg/mL proteinase K in PBS at 37 °C. Digoxigenin-labeled LNA probes suspended in hybridization buffer (50% formamide) were denatured at 95 °C for 2 min. Hybridization was performed at 60 °C overnight. After hybridization, samples were washed in 5 × SSC (saline–sodium citrate buffer) for 15 min, and then 2 × 30 min in 0.2 × SSC at 60 °C. After a 10-min PBS wash, samples were equilibrated in 10% goat serum-containing blocking buffer (0.5% blocking reagent (Roche, Budapest, Hungary) in PBS containing 0.1% Tween 20) for 1 h at RT and then incubated with anti-DIG alkaline phosphatase (AP) conjugate (Roche Diagnostics, Manheim, Germany) diluted 1:500 with blocking solution for 1 h. After antibody incubation, samples were washed in PBS containing 0.1% Tween-20 for 2 × 10 min and in PBS for 2 × 10 min. Coloring reactions were performed overnight with BM purple AP substrate (Roche Diagnostics, Manheim, Germany). Slides were mounted with Glycergel (Dako Denmark A/S, Glostrup, Denmark). Pictures were taken with the use of a Zeiss AxioImager fluorescent light microscope (Carl Zeiss MicroImaging, Thornwood, NY, USA) fitted with a PixeLINK CCD camera (PixeLINK, Ottawa, ON, Canada).

### 3.3. *In Vitro* Binding Assay

These experiments were conducted in accordance with the instruction manual of the Dual TRAP™ RNP Purification KIT (Cytostore Inc., Calgary, AB, Canada). The flow chart of the procedure is shown in Figure 2B. RNA template preparation for the binding assay was carried out as follows: genomic DNA was isolated from healthy human donor blood with a Perfect gDNA Blood Mini kit (Eppendorf, Mannenheim, Germany) in accordance with the manufacturer’s protocol. PCR amplification of the template region of the PRINS gene was performed with the following protocol: a denaturing step at 94 °C for 5 min, 30 cycles of denaturation at 95 °C for 30 s, annealing at 55 °C for 30 s, extension at 72 °C for 30 s and one cycle of terminal extension at 72 °C for 2 min. The sequences of the EcoRI-AK676 5′ FWD (5′ GGA ATT CTC CGT CTT AAA GGA AAA AAA TTT CTG 3′) and Bam-AK715 3′ REV (5′ CGG GAT CCT AGT CCC TCT CTC TGA TTT ATT G 3′) primers used for this PCR reaction The PRINS PCR amplicon was cloned into the EcoRI and BamHI site of the pPTRAP V5 vector (Cytostore Inc., Calgary, AB, Canada). Transcription was performed on the the pTRAP V5 vector linearized with the XhoI restriction enzyme (Sigma Aldrich, St. Louis, MO, USA). The efficiency of transcription was tested on 10% PAGE. We used the tagged RNA directly (with the reaction mixture) without further purification in the RNA/protein co-incubation and purification procedure.

The *in vitro* transcribed RNA (PRINS AK676 sequence) was added to the tobramycin affinity matrix, and co-incubated with cellular extracts (HaCaT cell extract *n* = 2; NHEK extract *n* = 1). The washing steps and the elution of the RNP particles were carried out as described in the users’ manual. The eluate (S) was transferred to the streptavidin affinity matrix. A second round of washings and elution were carried out (elute F_3_). Control elutes were obtained from both the tobramycin (T_end_) and the streptavidin (S_end_) columns, which did not bind the *in vitro* transcribed PRINS sequence: the cellular extracts flowed through them. In general, we carefully followed the instructions in the manual of the RNP purification kit,; however, some modifications were made as follows: during the purification of the PRINS RNA/protein complex, when the pre-binding of RNA transcripts to the 1st affinity matrix was performed, the *in vitro* transcribed RNA was incubated with RNAse inhibitor in Vial A for 60 min. Co-incubation of the cell extract with the *in vitro* transcribed RNA on the column was carried out overnight and this was followed by a two-step (2 × 30) elution.

Proteins in the elutes were separated on 7.5% SDS-PAGE and stained with Coomassie Brilliant Blue. As marker we, used Kaleidoscope prestained standards (Bio-Rad Laboratories, Cressier, Switzerland) containing seven individually color-coded proteins. The bands of interest were cut out and identified by MALDI-TOF mass spectrometry.

### 3.4. Real-Time RT-PCR

Total RNA was isolated from cells through the use of TRIzol TM Reagent (Invitrogene Corp., Carlsbad, CA, USA), following the manufacturer’s instructions. cDNA was synthesized from 1 μg total RNA with the iScript cDNA Synthesis Kit (Bio-Rad Laboratories, Hercules, CA, USA). Real-time RT-PCR experiments were carried out with the Universal Probe Library system (F. Hoffmann-La Roche AG, Basel, Switzerland). The PRINS primers were FWD: GAGGCCAGCAGTTTCTACAG and REV: AGGGACAACCACATCAAAGC and the Cy5-labeled CCTTCATCTCACACACCTACGCAG probe; NPM primers were FWD: GGAGGAGGATGTGAAACTCTTAAG and REV: CTCTTCATCATCATCGTCATCATC and the FAM-labeled probe was ATATCTGGAAAGCGGTCTGCCC; each probe was purchased from Integrated DNA Technologies Inc., Coralville, IA, USA. PCR assays were performed with an iQ5 Real-Time PCR Detection Machine (Bio-Rad). The relative mRNA levels were calculated by the ΔΔCt method [52]. The expression of each gene was normalized to the 18S ribosomal RNA gene.

### 3.5. Western Blot Analysis

During Western blot analysis, equal amounts of proteins were separated on 10% SDS-PAGE gel, transferred to Pure Nitrocellulose Membrane (Bio-Rad Laboratories) and immunoblotted with anti-NPM (Sigma Aldrich, St. Louis, MO, USA) antibody diluted 1:500. Anti-mouse IgG alkaline phosphate conjugate (Sigma Aldrich, St. Louis, MO, USA) was used as a secondary antibody, and signals were visualized with Sigma Fast TM BCIP/NBT (Sigma Aldrich, St. Louis, MO, USA). A-Actin was used as a loading control; α-actin-specific antibody was purchased from Sigma.

### 3.6. Immunohistochemistry

Paraffin-embedded tissues of healthy, involved and uninvolved psoriatic skin samples measuring 5 mm × 10 mm × 4 mm were obtained for NPM immunohistochemistry. The fixative was 4% buffered formaldehyde for 24 h. The tissue block was subjected to paraffin embedding and 4-μm-thick sections were placed on silanized slides, dewaxed in xylene for 3 × 5 min and rehydrated in decreasing concentrations of ethanol and methanol for 2 × 3 min and 2 × 2 min, respectively. Tissue endogenous peroxidase was blocked in a mixture of 90 mL of methanol and 3 mL of H_2_O_2_ for 5 min. For nonspecific antigen blocking, a mixture of 1% BSA and 0.1% Azide was used for 10 min. After nonspecific antigen blocking, sections were incubated with anti-NPM mouse monoclonal IgG antibody (Sigma Aldrich, St. Louis, MO, USA). The antibody was used in a dilution of 1:25 in LabVision Solution® (LabVision Corporation, Fremont, CA, USA) overnight.

For more sensitive reactions, HRP polymer (Envision^®^ system; Dako, Denmark) was applied for 30 min. The intensity of the DAB reaction (RealEnvision^®^ system; Dako, Denmark) was controlled under a light microscope. Sections were counterstained with hematoxylin for 1 min.

### 3.7. Gene-Specific Silencing

Gene-specific silencing of PRINS was performed with a vector-based method described previously [13]. The most effective PRINS silencing was achieved in supplement-free HPV-Ker cell cultures at ~70% confluency, and the transient transfection with the *in vivo* pSilencer™ 2.1-U6 hygro vector (Ambion Inc., Austin, TX, USA) was therefore carried out with these cells. The siRNA sequence targeting PRINS gene silencing was as follows: AK696, TTTCTGGAATGATGTCCAA. The scrambled sequence (SC1313) was used as control: AACTTTATCTCGGATCTAT. Cells were transiently transfected with plasmids following an X-tremeGENE 9 DNA transfection protocol, as described by the manufacturer (Roche Diagnostics, Mannheim, Germany). The transfection efficiency was on average 85%, as checked with a GFP reporter construct (Lonza, Basel, Switzerland). The effectiveness of the silencing was measured by RT-PCR (Figure S4).

### 3.8. Immunocytochemistry

Control and UV-treated HPV-Ker cells and NHEK cells were trypsinized and harvested by centrifugation, and resuspended in PBS. One hundred thousand cells were centrifuged onto a slide using a cytocentrifuge (Cytopro™, Wescor, Logan, UT, USA) and dried overnight at RT. The slides were fixed in 2% paraformaldehyde for 20 min at RT, and then washed in TBS. Nonspecific antigens were blocked for 30 min at RT in 1% goat serum containing 0.5% BSA-TBS. Slides were incubated overnight at 4 °C with anti-NPM monoclonal antibody (Sigma Aldrich, St. Louis, MO, USA; 1:500) in 0.5% BSA-TBS. Anti-mouse Alexa Fluor 546 goat anti-mouse IgG secondary antibody solutions (Sigma Aldrich, St. Louis, MO, USA; 1:500) were applied for 3 h at RT. Cell nuclei were counterstained with DAPI (Sigma Aldrich, St. Louis, MO, USA, 1:100) and mounted with Fluoromont-G (Southern Biotech, Birmingham, AL, USA). Pictures were taken with the aid of a Zeiss AxioImager fluorescent light microscope (Carl Zeiss MicroImaging, Thornwood, NY, USA) fitted with a PixeLINK CCD camera (PixeLINK, Ottawa, ON, Canada).

For the semiquantitative analysis of the intracellular localization of NPM (nucleolar or nuclear), at least 25 fields of view were counted per each group by two independent examiners. Cells were sorted and counted by observing the localization of NPM immunostaining in the nucleolus or in the nucleoplasm.

### 3.9. *In Silico* Prediction of PRINS-Binding Proteins

*In silico* prediction of the putative PRINS-binding proteins was carried out with the MatBase program (www.genomatix.de).

## 4. Conclusions

We have demonstrated here that the ncRNA PRINS interacts physically and functionally with the chaperone molecule NPM. The crucial regulatory roles of NPM in cell proliferation and cellular stress response have already been demonstrated [53–55] and studied in detail. In agreement with previous findings, we revealed that NPM is associated with proliferation of the keratinocytes. The data we have reported here suggest that PRINS might be part of a regulatory complex formed with NPM. The resulting complex may play a role in the regulation of proliferation and differentiation and in the stress response of cells. We hypothesize that the abnormal functioning of this complex contributes to the pathogenesis of both malignant and benign hyperproliferative diseases, such as psoriasis.

## Supplementary Information
