# Supplementary material for: Expression and Functional Studies on the Noncoding RNA, PRINS
Source: Int J Mol Sci. 2012 Dec 21;14(1):205–25. doi: 10.3390/ijms14010205 (PMC3565259; doi:10.3390/ijms14010205)
Supplement: Supplementary file 1 [file ijms-14-00205-s001.pdf]

Supplementary Information

Figure S1. The proliferation/differentiation state of keratinocytes. The *in vitro* expression of NPM was followed in 3rd passage keratinocytes (See Figure 5). The 0 h samples were taken from subconfluent cultures. The process of differentiation was followed by the increase of the differentiation marker keratin 10 and the decrease of the proliferation-related gene, alpha5 integrin.

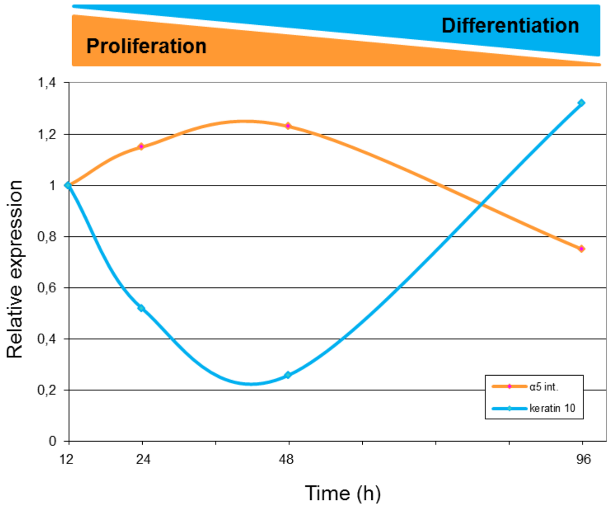

Figure S2. UV-B induced NPM trafficking in NHEKs. Unirradiated and UV-B-irradiated (312 nm, 40 mJ/cm^2) NHEKs were followed for the indicated periods of time, fixed and immunostained for NPM. DNA was stained with DAPI.

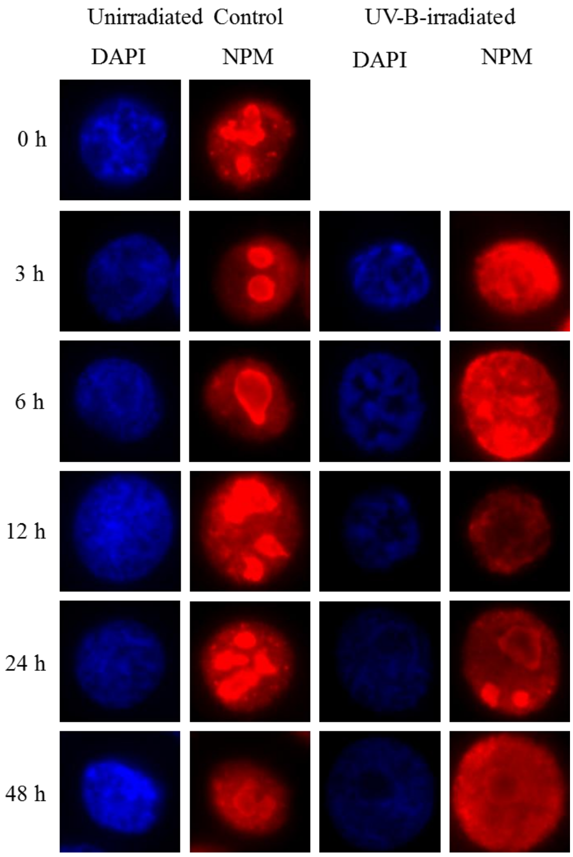

Figure S3. The effectiveness of PRINS gene-specific silencing and its effect on NPM expression. HPV-Ker cells transfected with a pSilencer vector expressing an siRNA specific for PRINS (AK696, *n* = 3) or a scrambled sequence (SC1313, *n* = 3) were cultured in supplement-free medium for 24 h. Data are indicated as fold expressions compared to empty vector-transfected cells (*n* = 3). Error bars shouw the SEM of the results of three independent experiments. (**A**) PRINS expression was determinded by real-time RT-PCR. (**B**) NPM expression was determinded by real-time RT-PCR.

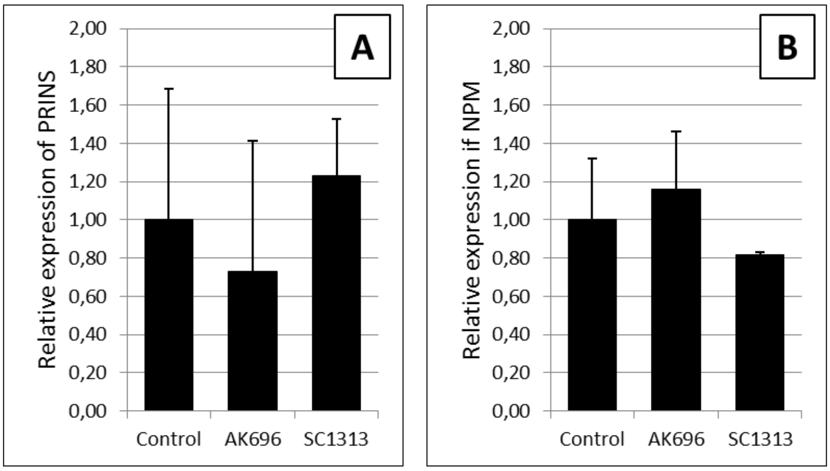

Figure S4. The effect of PRINS gene-specific silencing on the intracellular shuttling of the NPM-GFP construct in HPV-Ker cells. HPV-Ker cells were transfected with an NPM-GFP chimeric construct. Next day the cells were transfected with the PRINS gene-specific silencing vector (AK696), or with the vector containing the scrambled sequence (SC1313), or with an empty vector as control. Twenty-four hours after transfection the unirradiated and the UV-B-irradiated (312 nm, 40 mJ/cm^2) HPV-Ker cells were followed for the indicated periods of time, fixed and stained with DAPI (blue).

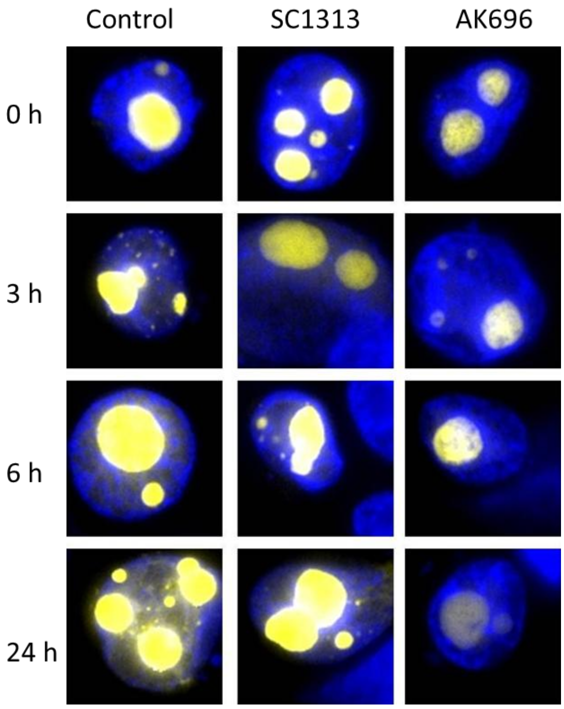

Table S1. *In silico* prediction of PRINS binding proteins.

**No. of nucleotide on PRINS sequence**		**Description of putative binding factor**
422	433	Krueppel-like C2H2 zinc finger factors hypermethylated in cancer
934	956	Peroxisome proliferative activated receptor homodimers
1233	1249	Olfactory associated zinc finger protein
1246	1258	GDNF-inducible zinc finger gene 1
1251	1263	C/EBP homologous protein (CHOP)
1327	1341	Sterol regulatory element binding proteins
1384	1394	D-abl DNA binding sites
1394	1406	GHF-1 pituitary specific pou domain transcription factor
1618	1626	AARE binding factors
1781	1795	Metal induced transcription factor
1786	1800	Sterol regulatory element binding proteins
1801	1813	OCT6 binding factor_astrocytes + glioblastoma cells
1832	1850	Microphthalmia transcription factor
1855	1893	General transcription factor IID, GTF2D
1976	1992	Carbohydrate response elements, consist of two E box motifs separated by 5 bp
2033	2043	ZF5 POZ domain zinc finger
2329	2341	Krueppel-like C2H2 zinc finger factors hypermethylated in cancer
2668	2680	Regulator of B-Cell IgH transcription
2687	2697	Calsenilin, presenilin binding protein, EF hand transcription factor
2987	2997	Calsenilin, presenilin binding protein, EF hand transcription factor
3117	3129	RP58 (ZFP 38) zinc finger protein
3145	3157	GDNF-inducible zinc finger gene 1
3236	3248	Y-box binding transcription factors, multifunctional proteins involved in transcriptional and translational regulation, mRNA splicing, DNA replication and repair
3274	3286	Farnesoid S-activated receptor response elements
3318	3334	Olfactory associated zinc finger protein
3461	3473	Regulator of B-Cell Ig H transcription

© 2013 by the authors; licensee MDPI, Basel, Switzerland. This article is an open access article distributed under the terms and conditions of the Creative Commons Attribution license (http://creativecommons.org/licenses/by/3.0/).
